# Safety and authenticity risks in heritage food preparation at different types of food service establishments: A case study of Saudi Arabia

**DOI:** 10.1016/j.heliyon.2023.e13042

**Published:** 2023-01-21

**Authors:** Mohammad Almansouri, Pieternel Luning, Majed Almuhanna, Ruud Verkerk

**Affiliations:** a Food Quality and Design Group, Department of Agrotechnology and Food Sciences, Wageningen University and Research, P.O. Box 17, 6700 AA, Wageningen, the Netherlands; b King Saud University, Riyadh, Saudi Arabia; c Ministry of Culture, Culinary Arts, Riyadh, Saudi Arabia

**Keywords:** Heritage food, Authenticity risks, Safety risks, Hospitality industry, Risk situations, Culinary professionals

## Abstract

In Saudi Arabia, tourism is becoming increasingly popular, and forms an essential element of Vision 2030. Accordingly, food service establishments (FSEs) including hotels, ordinary restaurants, heritage restaurants and productive families (i.e., home-based catering) provide heritage cuisine to tourists. This study aimed to assess the authenticity and safety risks associated with the production of heritage food dishes in different FSEs. An online questionnaire was administered in Saudi Arabia, and a total of 85 culinary professionals from different FSEs responded. The culinary professionals were requested to provide opinions on the frequency of food safety and authenticity risk situations at their FSEs, using a five-point Likert scale. The results indicate that most food safety risk situations occur less frequently in hotels because of strict food safety management systems. In contrast, food safety risk situations are more frequent in ordinary and heritage restaurants, particularly in the absence of personal hygiene requirements. In productive families, most food safety risk situations occur because there are no control systems or inspections. Authenticity risks occur less frequently in productive families and heritage restaurants than in other FSEs. Hotels often/always face authenticity risk situations, such as cooking of heritage dishes by non-Saudi culinary professionals and the use of modern equipment. Ordinary restaurants face the highest risk, mostly because of the limited knowledge and skills of the cooks. Overall, this study provides the first insight into the occurrence of possible safety and authenticity risk situations during the preparation of heritage dishes; this information may contribute to improve the production of safe and authentic heritage dishes in the hospitality industry for tourists and local people.

## 1 Introduction

Recently, heritage cuisine, which is a part of the identity and culture of a destination, has gained increasing interest [1,2]. Heritage cuisine has become an attraction for tourists expecting rare or exceptional culinary experiences [[3], [4], [5], [6]]. Tourist demand for heritage foods and culinary traditions may help increase awareness of their value and even promote their preservation. Heritage food involves intangible elements such as dining etiquette, service style, specialty ingredients, and preparation. Previous studies have developed and validated a heritage food concept encompassing three main dimensions: legacy, people, and place, as well as factors that can compromise their safety or authenticity [7]. Various risk factors, such as lack of adequate knowledge of chefs and scarcity of ingredients, can specifically compromise the authenticity of heritage food; further, food safety risk factors are applicable to foods typically prepared in food service establishments (FSEs) [8].

Heritage or traditional dishes are prepared in different types of FSEs such as hotels, ordinary restaurants, and specific heritage food restaurants [[9], [10], [11], [12]]. These establishments differ in their clients, size, menus, and management [13]. Hotels are typically used by tourists, with a growing interest in heritage food; thus, hotels increasingly serve heritage food on their menus [14]. Ordinary restaurants usually serve heritage dishes mainly to the local population, but also to tourists [15]. [16] noted that visitors of heritage restaurants were significantly more motivated to understand the local history and culture, and more open a ‘new eating experience’.

In some countries, productive families or home-based catering represent another type of FSE in the catering business [17,18]; these also prepare heritage food. In Saudi Arabia, a productive family (home-based catering) is defined as a family of one or more individuals residing in one house and engage in preparing foods for local sale including heritage dishes [19]. For example, in Saudi Arabia, it is quite common for women to prepare heritage dishes in their homes for catering wedding parties or large dinners. They are quite successful because they offer more personalised services at a relatively low price compared with those of hotels and restaurants that prepare similar heritage foods [18]. Thus, different types of FSEs serve heritage foods, but their clients, size, menus, and management differ substantially.

These differences in the FSE type and client expectations can also influence the authenticity and safety of heritage foods. For example, authentic heritage food dishes served to the local population could differ from the dishes served to tourists. The challenge of adjusting authentic recipes to satisfy tourist demands may lead to authenticity risks [8,20,21]. For instance, in a study of the Bosnian cuisine [22], found there was a poor style of presentation in many restaurants serving tourists [23]. Food safety risks may also differ because of the different characteristics of FSEs [13]. In general, the food safety management systems (FSMS) in FSEs differ substantially from those of the food industry, as FSEs need to prepare a large number of meals that must be partly prepared in advance, often in the same area; further, the number of clients is not known beforehand and the workers stand for long periods performing repetitive activities [[24], [25], [26], [27]]. Several studies have reported that food safety problems occur within the catering industry because of staff and FSE characteristics, such as education levels, large numbers of complex meals, food provision to numerous vulnerable consumers, variety of operations, and limited knowledge of the staff concerning food safety [[28], [29], [30]]. They also suggest a relationship between the catering business type and food illness outbreaks. Food safety management systems are based on implementing good hygiene practices (GHP) and hazard analysis and critical control points (HACCP); therefore, these differ for each FSE. Most studies have mainly focused on the food safety of common meals served in FSEs. To the best of our knowledge, differences in the authenticity and safety risk factors of FSEs serving heritage food remain to be studied.

Therefore, this study aimed to assess the safety and authenticity risks associated with the production of heritage food dishes in different FSEs. This study was performed in Saudi Arabia because of its growing tourism industry, and the relative novelty of the hospitality industry serving heritage food to tourists. Safety risks are expected to be higher in productive families than in other FSE types, whereas authenticity risks are expected to be higher in hotels.

## 2 Materials and methods

### 2.1 Questionnaire design

The questionnaire was designed to gain insights into food authenticity and safety risk situations as perceived by culinary professionals from different FSEs. The questionnaire comprised four sections and was designed based on [31]. The first section included general questions to typify the respondents (e.g., city, kind of dishes they cook, and how they learned to prepare heritage food), and their definition of the heritage food concept. The second section reflected the characteristics of their FSEs. The third and fourth sections included questions related to risk situations that could compromise the authenticity or safety of heritage food preparations, respectively. The closing section included questions to typify the respondent personal characteristics, such as age, level of education, and experience in cooking heritage food. The culinary professionals were requested to provide their opinions on how frequently food safety and authenticity risk situations occur at their FSEs, using a five-point Likert scale (always, often, sometimes, rarely, and never). The questionnaire was translated into Arabic because the respondents were more familiar with their native language than with English. The questionnaire was pretested by two culinary professionals to determine its readability and comprehensibility, after which minor changes were made before the survey was administered. Supplementary Material 1 contains the detailed questionnaire.

### 2.2 Selection of culinary professionals

Culinary professionals were selected according to the following criteria: (1) more than five years of experience working in the hospitality industry, (2) more than five years of experience cooking various heritage food dishes, (3) local nationality (Saudi), and (4) involvement in the production of heritage food dishes; (5) particularly for productive families, educational background (bachelor's degree) was considered because it enabled them to understand the survey contents. Culinary professionals (hotels, ordinary restaurants, and heritage restaurants) were selected based on the available information about FSEs in Saudi Arabia and through authors' networks. They were asked to complete an online survey using Qualtrics, an online survey platform allowing survey design, distribution, and response analysis from a single convenient online location. Qualtrics is widely used in academic research and is a user-friendly online platform including a large array of question types; further, it can translate a survey into multiple languages [32]. The final sample comprised 85 respondents.

### 2.3 Conducting the survey

Potential respondents were informed about the characteristics and aim of the study, data anonymisation and their confidentiality. Subsequently they were inquired for their willingness to participate. Participating respondents provided their oral consent. The survey took 10–15 min to complete. We checked all the surveys to determine whether the answers were fully completed. For productive families, we contacted the respondents personally to check whether all questions were clear and understandable. If the questions were unclear, additional information was provided.

### 2.4 Data analysis

All data were entered into Excel for translation into English. All statistical analyses were then performed using the IBM SPSS Statistics Version 25. Descriptive statistics were performed to determine the frequency of the respondent responses regarding their demographic information and FSE characteristics. Cross-tabulation analysis (crosstab) was performed to understand the relationship between FSEs and each food safety and authenticity risk situation to determine the number of culinary professionals who indicated the occurrence of these situations. The analysis of risk situation occurrence was based on whether half or more than half of the culinary professionals (depending on the FSE) indicated always, often, sometimes, rarely, or never.

## 3 Results and discussion

### 3.1 Characteristics of culinary professionals and FSEs

Table 1 shows the characteristics of culinary professionals in FSEs (i.e., hotels, ordinary restaurants, heritage restaurants, and productive families). Most culinary professionals working in hotels, ordinary restaurants, and heritage restaurants were men, whereas those in productive families were all women. The majority of culinary professionals were between 30 and 49 years of age. In hotels and ordinary restaurants, six cooks completed secondary schooling at the highest level, whereas the others had a subject diploma in food (n = 10, n = 5, respectively), bachelor's degree (n = 5, n = 3, respectively), or postgraduate degree or higher (n = 4, n = 6, respectively). Most respondents from heritage restaurants and productive families had a diploma (n = 6, n = 4, respectively) or bachelor's degree (n = 9, n = 13, respectively), and a few completed secondary school or below. Half of the culinary professionals in hotels, ordinary restaurants, and heritage restaurants had between one and nine years of experience in the hospitality industry, whereas in productive families, half of the culinary professionals had no experience in the hospitality industry. These results are aligned with those of [33,34], who found that most culinary professionals in Saudi Arabia have experience ranging between 1 and 10 years in the hospitality industry. This could be attributed to the relative novelty of the tourism sector and is a promising part of Saudi Arabia's Vision 2030 which envisages the tourism sector as a significant contributor to job creation and a source for developing more skilled workers in this sector [35].

**Table 1:** Characteristics of culinary professionals in the foodservice establishments.

Characteristics of ..culinary professionals/cooks	Hotels N = 25	Ordinary Restaurant N = 20	Heritage Restaurants N = 20	Productive Families N = 20
Gender				
Male	13	14	10	*
Female	12	6	10	20
**Age**				
20–29	5	4	2	1
30–39	10	7	7	13
40–49	9	8	7	3
50–59	1	1	3	3
60 or more	*	*	1	*
**Highest education level**				
Below secondary school	*	*	3	1
Secondary school	6	6	2	2
Subject diploma in food	10	5	6	4
Bachelor	5	3	9	13
Postgraduate degree or above	4	6	*	*
**Experience in the hospitality industry** (Years)				
No experience	*	*	*	10
1–9	12	15	12	7
10–19	7	5	4	3
20–29	6	*	1	*
30–39	*	*	3	*
**Experience in cooking HF dishes** (Years)				
1–9	11	9	12	8
10–19	9	6	4	6
20–29	4	3	1	1
30–39	1	2	3	5
**Learning to cook HF dishes**				
Parents/grandparents	18	16	16	17
Relatives	8	5	8	6
Social Media	3	7	4	4
Book	2	2	1	1
Work	10	1	6	2
**Food safety training**				
No food safety training	6	10	10	15
Basic food safety training in a restaurant	9	8	8	5
Official food safety training (HACCP, ISO22000, others).	13	2	3	2
Food safety courses in education (university).	7	1	2	1

Specific food safety training was provided to the majority of culinary professionals in hotels (n = 13 official training, n = 9 basic, and n = 7 specific courses), whereas most culinary professionals in other FSEs only underwent basic food safety training or no training at all. Most culinary professionals learned to cook heritage food from their parents or grandparents. In Saudi Arabia, training of cooks in the hospitality industry began in 2009 [36]. Currently, training has become an essential requirement in hotels and restaurants [37]. According to Ref. [38], ongoing training of food handlers and conveying science-based information on food safety risks are essential for enhancing their awareness, positive attitudes, and compliance with food safety requirements and operating procedures.

Table 2 shows that productive families are typically family-owned businesses, whereas the other FSEs, were privately owned, part of a chain, or family-owned. The productive families employed only Saudi Arabian workers, whereas the other FSEs also employed workers of other nationalities. Regarding food safety guidelines and standards, hotels implemented various standards, such as hazard analysis and critical control points (HACCP) (n = 20), good hygiene practices (GHP) (n = 10), ISO 22000 (n = 13), and ISO9001 (n = 10); 12 and 8 hotels had an ISO2200 and ISO9001 certificate, respectively. HACCP and GHP were also implemented by ordinary (n = 9 and n = 2, respectively) and heritage restaurants (n = 8 and n = 8, respectively), whereas productive families did not implement any guideline/code. In Saudi Arabia, in 2009, the municipalities issued regulatory guides for hygiene and sanitation and HACCP implementation in FSEs [36,39] but no specific guidelines or rules were set for productive families. In the United Arab Emirates (UAE), a neighbouring country of Saudi Arabia, domestic workers are often not governed by regulations/regular food safety inspections or compulsory training [40]. Saudi Arabia and UAE are two Middle Eastern countries with the same culture for domestic working. In hotels, compliance with food safety regulations is typically evaluated through unannounced inspections by public authorities (n = 20), announced third party audits (n = 10), and internal audits (n = 17). In ordinary and heritage restaurants, this is typically done through annual inspections by public authorities (n = 8 and n = 10, respectively), unannounced inspections by public authorities (n = 12 and n = 10, respectively), and internal audits (n = 12 and n = 9, respectively). Although commercial FSEs are regularly inspected to ensure implementation of FSMS, HACCP programs, risk control procedures, and monitoring systems, productive families have yet to be included in any official inspection program and they do not typically conduct any kind of internal audit. Specific parameters are monitored by the appointed inspectors, including FSE maintenance, employee hygiene, and storage control [41]. In case of non-compliance, the municipality commonly gives a warning (n = 20, n = 15, and n = 15, respectively), conducts follow up inspections (n = 12, n = 9, and n = 15, respectively) and imposes fines (n = 17, n = 12, and n = 13, respectively) (Table 2). For instance, in 2013, more than 350 restaurants were temporarily closed and fined by Riyadh Province Municipality (RPM) after an intensive campaign against establishments that violated food safety protocols [37].

**Table 2:** The characteristics of different foodservice establishments.

Characteristics of FSE	Hotels N = 25	Ordinary Restaurants N = 20	Heritage Restaurants N = 20	Productive Families N = 20
**Characterizing the FSE**				
Private owner	3	11	8	a
Part of chain	10	4	6	a
Family owner	12	5	6	20
**Native Saudi Arabian workers**				
None	2	8	4	a
A few	15	10	12	a
Some	5	1	2	a
Most	3	1	2	a
All are Saudi workers	a	a	a	20
**Target clients**				
Regional residents	17	19	16	20
International residents	9	2	9	5
Local visitors	14	7	7	7
International visitors	16	3	9	3
**Regulations/guidelines/standards of FSE**				
HACCP	20	9	8	N/A
GHP (Good Hygiene Practices)	10	2	8	N/A
ISO22000	13	2	5	N/A
ISO9001	10	5	1	N/A
**Certificate of FSE**				
ISO22000	12	2	4	N/A
ISO9001	8	4	1	N/A
**Evaluating compliance with food safety regulation**				
Annual inspection by public authorities	5	8	10	N/A
Unannounced inspection by public authorities	20	12	12	N/A
Inspection in case of problems by public authorities	4	1	7	N/A
Announced third-party audits to check compliance with the standards	10	4	2	N/A
Unannounced third-party audits to check compliance with the standards	7	1	3	N/A
Internal audit	17	12	9	N/A
**Measures for non-compliance to food safety regulations**				
Warning	20	15	15	N/A
Follow up inspection	12	9	15	N/A
Fine	17	12	13	N/A
Suspension	9	6	9	N/A
End of licenses	5	1	4	N/A
Closure of establishment	6	4	6	N/A
**Measures for non-compliance with the private standards (ISO22000 and ISO9001)**				
Warning	13	9	10	N/A
Following-up audits	12	10	7	N/A
Suspension	11	6	5	N/A
Certificate not extended	9	3	3	N/A

a N/A: Not Applicable.

### 3.2 Dimensions of the heritage food concept

The concept of heritage food is relatively new, and there is no clear consensus yet. Therefore, we investigated culinary professionals’ perceptions of the three previously identified heritage food dimensions: legacy, people, and place [7]. The legacy dimension indicates that the heritage food is passed from the past generation to the present and is part of the origin country [42]. The people dimension is related to a specific ethnic group with specific knowledge of food preparation, which originates from a unique and distinct culture [43,44]. Heritage food is particularly related to place because it refers to a specific destination where the raw materials for preparing the food and drinks are produced locally [45]. Most culinary professionals from FSEs (strongly) agreed on all three dimensions, and their importance in the production of heritage foods. Only a few culinary professionals disputed the relevance of these dimensions. These findings further confirm the relevance of these dimensions, which have also been validated by experts in another study [8].

### 3.3 Food safety risk situations in the production of heritage food

Table 3 shows the number of culinary professionals who indicated how frequently food safety risk situations occur in the production of heritage food in their hotels, ordinary restaurants, heritage restaurants, or productive families, along with their mode scores. Food safety risk situations were divided into four categories: receiving raw ingredients, storage, processing (preparing and cooking), and personal hygiene. Overall, for hotels, a mode score of three was obtained for all food safety risk situations, indicating that the highest number of hotel culinary professionals responded that these situations rarely/never occurred. In contrast, for ordinary and heritage food restaurants, personal hygiene-related risk situations mostly had a mode score of one, indicating that the highest number of culinary professionals in these restaurants responded that these situations occur often/always. Productive families showed a mode score of one for more than half of the safety risk situations, related to receiving raw materials (situations 1 and 2), storage (3, 5), processing (8, 9), and personal hygiene (13, 15, 16, 17).

**Table 3:**
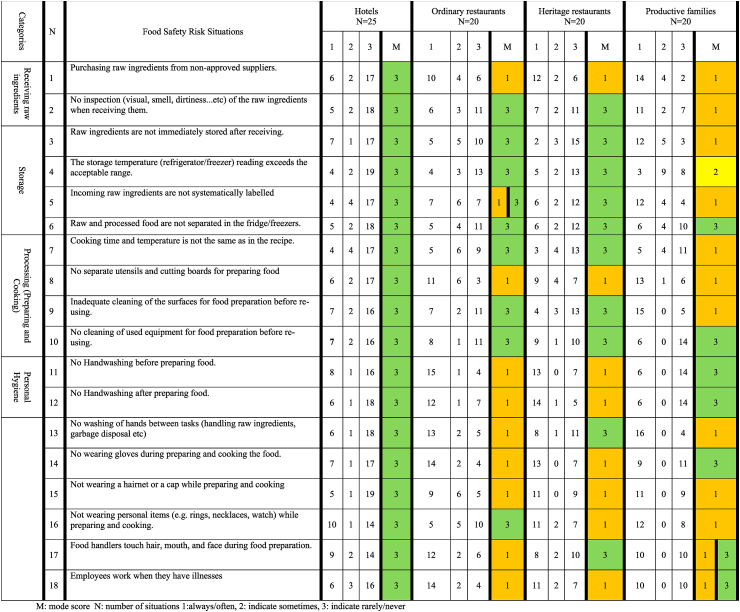
Occurrence of particular food safety risk situations upon production of heritage food dishes as assessed by culinary professionals in various food service establishments.

The relatively low occurrence of food safety risk situations in hotels could be attributed to the implementation of the GHP and/or HACCP guidelines and/or the ISO2000 standard in their FSMS. Furthermore, these systems are mostly evaluated through unannounced inspections by public authorities and internal audits (Table 2) [46]. investigated the role of FSMSs in five-star hotels and found a significant improvement in food safety. They attributed this improvement to better personal hygiene procedures, improved environmental hygiene, more adequate food storage conditions, and the use of sanitary measures to prevent cross-contamination. Similarly [47],concluded that HACCP-based FSMS improved food quality and safety in hotels. Nevertheless, Table 3 shows that between four and eight culinary professionals from hotels indicated that several food safety risk situations (situations 1 to 18) occur often or always. Thus, although hotels implement food safety guidelines and standards, it can be challenging for staff to implement them accurately and obey all the rules, as reported in various studies [13,48,49]. In a study of 265 food handlers and 32 culinary professionals and managers from hotels in Brazil by Ref. [49], most food handlers had good knowledge, attitudes, and practices that were appropriate for personal hygiene. Nevertheless, some non-conformities were identified, including failure to use disposable gloves when handling or distributing food, and tasting food with their hands.

Regarding food safety risk situations related to storage, Table 3 shows that many culinary professionals in ordinary (10–13) and heritage restaurants (12–15) indicated that they rarely or never compromised on storage requirements (situations 3, 4, and 6). In contrast, more than half of the culinary professionals in productive families indicated that they did not often/always have the required storage conditions. This could be explained by the fact that they commonly purchase raw materials from the local market (usually non-approved suppliers) and immediately prepare heritage food dishes without storing any ingredients or dishes in a refrigerator [50]. observed that Saudi women commonly buy fresh ingredients for their traditional diet, which are then prepared and cooked immediately to maintain authentic taste. However, there is a potential safety risk in keeping the ingredients or prepared dishes outside the storage conditions before delivery to clients. Owing to the characteristic high temperatures, especially in the Middle East, fresh foods (mostly of animal origin) are prone to rapid spoilage, quality degradation, and most importantly, hazards related to foodborne infectious diseases [38].

Regarding personal hygiene, between 10 and 15 culinary professionals from ordinary and heritage restaurants specified that they often or always compromised on various personal hygiene requirements (Table 3). The relatively high occurrence of unsafe personal hygiene situations in ordinary and heritage restaurants could be attributed to non-implementation of strict FSMS based on GHP and HACCP by most of these FSEs (Table 2). According to Ref. [37], restaurants in Saudi Arabia were fined and closed for violations such as poor personal hygiene, working without valid health certificates, and workers with visible signs of disease.

In productive families, more than half of the culinary professionals indicated that they rarely/never compromised on the requirements of handwashing before and after preparing and cooking (situations 11, 12). Interestingly, for all other personal hygiene-related risk situations, culinary professionals indicated either that they often/always or rarely/never compromised these requirements. In a study of 1490 Saudi women by Ref. [51], women aged 26 years and older, with marriage experience, and with children had significantly higher food safety knowledge than that of young, single women and women without children. Moreover, women with higher education levels and those employed in health-related professions showed significantly higher knowledge and practice scores than others. This suggests that the structure of productive families could play a role in having good personal hygiene practices.

### 3.4 Food authenticity risk situations in the production of heritage food

Table 4 shows the number of culinary professionals indicating how frequently food authenticity risk situations occur during the production of heritage food in their hotels, ordinary restaurants, heritage restaurants, or productive families, and the mode scores. Overall, for heritage restaurants, Table 4 shows that for all food authenticity risk situations, the mode score was three, indicating that the highest number of culinary professionals responded that these situations rarely/never occurred. These restaurants are dedicated to heritage food and aim to preserve and maintain the authentic character of heritage dishes [52]. However, a substantial number of culinary professionals reported that authenticity risk situations sometimes, often, or always occur. Heritage restaurants (nine culinary professionals), as well as ordinary restaurants (13 culinary professionals), were found to not always follow recipes because of the limited knowledge and skills of the culinary professionals/cooks. This is a major issue in Saudi Arabia, as sufficient culinary professionals of Saudi nationality are not available to work at these restaurants. In Saudi Arabia, food service outlets, such as restaurants, favour employing foreign workers because of their willingness to work for low wages [53].

**Table 4:**
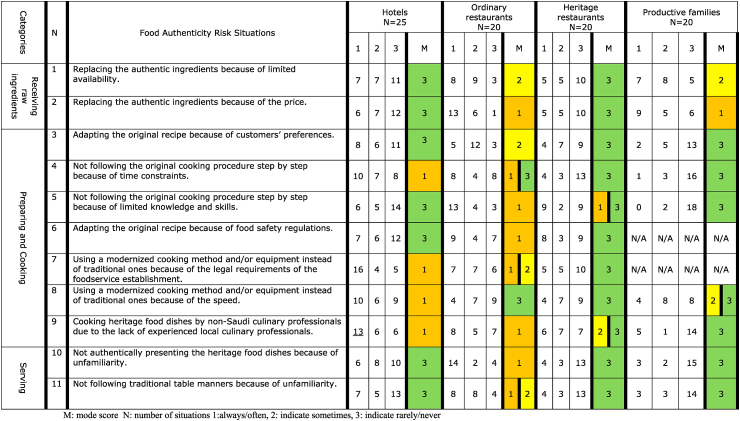
Occurrence of particular food authenticity risk situations upon production of heritage food dishes as assessed by culinary professionals in various foodservice establishments.

For productive families, the mode score was three for more than half of the authenticity risk situations related to preparing and cooking (situations 3, 4, 5, 8, 9), and serving (10, 11) (Table 4). However, two food authenticity risk situations, related to receiving raw materials (1, 2) were found to occur sometimes or often/always, such as replacing authentic ingredients because of limited availability and price. This could be explained by the fact that productive families have relatively low incomes [17], which limits their budget for buying ingredients; therefore, they may replace these with cheaper substitutes, which may influence the authentic taste [2].

For hotels as well, more than half of the food authenticity risk situations showed a mode score of three. These situations are linked to receiving raw ingredients (1, 2), preparing and cooking (3, 5, 6), and serving (10, 11). Between 13 and 16 culinary professionals indicated that they often/always use modernised equipment because of the requirements of their FSE. This can be explained by the fact that hotels have high standards for food safety, which do not always allow the use of traditional equipment [38]. Moreover, several culinary professionals (13) from hotels indicated that heritage food dishes are often/always cooked by non-Saudi culinary professionals because of the lack of experienced local culinary professionals, mainly attributed to the focus on an international workforce, which is typical for hotels. However, this might influence authenticity because foreign food handlers or culinary professionals could be influenced by their own cultural backgrounds [54,55], which could affect the preparation practices of heritage food.

For ordinary restaurants, most authenticity risk situations showed a mode score of one, indicating that the highest number of culinary professionals responded that these situations occur often/always. These authenticity risk situations were related to receiving raw ingredients (2), preparing and cooking (4, 5, 6, 7, 9), and serving (10, 11) (Table 4). The most authenticity risk situations indicated by the majority of culinary professionals were related to replacing the ingredients because of price and not authentically presenting the heritage dishes because of unfamiliarity. Ordinary restaurants in Saudi Arabia usually provide different types of dishes, and are not purely focused on heritage dishes; thus, they may not serve authentic local cuisine [15].

## 4 Implications to ensure food safety and authenticity of heritage food

Food safety risks are potentially higher in productive families and ordinary restaurants than in hotels. In hotels, food safety risk situations do not vary significantly, as they commonly have a FSMS in place, which is regularly inspected or internally audited. However, hotels still face challenges in fulfilling all hygiene requirements. For ordinary and heritage restaurants, risk situations do not differ significantly, and they mainly occur with personal hygiene issues, which may imply a risk for clients. For instance, an outbreak occurred at a traditional wedding in Saudi Arabia after food was delivered from a traditional restaurant, wherein 88 of 238 guests developed gastroenteritis [56]. Unhygienic practices during food preparation were discovered, including cooking with bare unclean hands and lack of handwashing. Moreover, after the food was delivered to the wedding, it was served traditionally, first to the males and then to the females, resulting in its exposure to room temperature for a long period during the celebration. Consequently, *Salmonella*, which was present in the heritage dish meat or rice (or both), multiplied to numbers sufficient to cause more infections in females than in males [56].

Based on our findings for productive families, food safety risk situations can occur at all stages of heritage food production. According to Ref. [57], homemade preparations and practices continue to be a part of daily life in the Middle East and Gulf regions, posing a risk of unhygienic safety practices including improper cooking, unhygienic food handling, and high ambient temperature [58]. Aconducted a study among Saudi Arabian women to assess food safety and hygiene awareness, behaviour, and practices. The findings showed that most women in the Eastern region (>90%) were well aware of food safety and washed their hands, cutting boards, knives, and plates. However, approximately 25% of female respondents considered that it was “safe” to keep hot/cold foods out of the refrigerator for more than 4 h, while 60% believed it “safe” to thaw frozen foods outside the refrigerator. Our study demonstrates that various food safety risk situations still occur often or frequently in these FSEs, together with a lack of formal inspections, which may imply risks for consumers eating heritage food prepared by productive families (Table 3).

Regarding authenticity, the differences in the occurrence of risk situations between the FSEs were less obvious. Overall, heritage restaurants and productive families revealed that fewer authentic risk situations occur often/always. The main differences between productive families and heritage restaurants are that all productive families are Saudi citizens with many years of experience in cooking heritage dishes, whereas heritage restaurants employ more international workers who lack the knowledge to prepare authentic dishes. This is an essential aspect of the heritage food dimension “people” [8]. [8] discussed that the non-native origin of culinary professionals could be a challenge in producing authentic dishes because heritage food is part of the legacy, culture, and identity of a region.

In hotels, the authenticity of heritage food dishes can be compromised mostly by time constraints, availability of local staff, and the legal requirements of the FSE. For example, most culinary professionals mentioned that using modernised equipment instead of traditional equipment because of legal requirements or speed mostly occurs in hotels (Table 4). The need for a quicker service for a wide variety of dishes to tourists may also force cooks to use alternative equipment or methods to speed up preparation [8]. Therefore, it might be challenging for local culinary professionals to safeguard the authenticity of dishes as well as satisfy tourist demands. These risk situations are particularly critical when the heritage dish recipes are complex and strictly require the use of traditional equipment to obtain authentic flavour, texture, and appearance. However, authenticity can still be safeguarded for other heritage dishes that are simpler and require less demanding equipment and recipes.

In ordinary restaurants, authenticity risk situations occur more often than in other FSEs because they do not strictly care for the authenticity of the heritage dish. According to Ref. [59], who conducted interviews with autochthones and expats in Saudi Arabia, restaurants rarely present a strictly local menu, which is always accompanied by other cuisines, such as Yemeni and Lebanese. Native people stressed that the food served in restaurants is mostly local, but not necessarily traditional, owing to the influence of cooking styles from other cultures. This may result in a lack of explicit representation of “traditional cuisine” through institutional and social channels, which erodes the authenticity of the culinary experience [59].

## 5 Conclusion

To our knowledge, this is the first study to assess the authenticity and safety risk situations in different types of FSEs involved in preparing heritage foods in Saudi Arabia. Our results indicate that in hotels, food safety risk situations occur less frequently than in other FSEs. In ordinary and heritage restaurants, food safety risk situations occur more frequently, particularly those related to personal hygiene requirements, which may have potential implications for the safety of heritage foods. Most food safety risk situations occur more frequently in productive families than in other FSEs. In contrast, in heritage restaurants and productive families, the fewest authenticity risk situations occur often/always. Ordinary restaurants have the most authenticity risk situations that occur often/always. Overall, this study indicates the importance of the economy, citizens' health, and protection of tourists in Saudi Arabia. Moreover, the insights provided by these findings can enhance the awareness of safety and authenticity risks in heritage food preparation and contribute to the production of safe and authentic heritage dishes for both locals and tourists in FSEs. Further research could lead to more in-depth insights on effects of preparation heritage foods on safety and authenticity risks in food service establishments. Hereby use of profound interviews with culinary professionals and on-site observations are useful methodologies.

## Author contribution statement

Mohammad Almansouri: Conceived and designed the experiments; Performed the experiments; Analyzed and interpreted the data; Wrote the paper.

Pieternel Luning: Conceived and designed the experiments; Analyzed and interpreted the data; Wrote the paper.

Majed Almuhanna: Performed the experiments; Analyzed and interpreted the data.

Ruud Verkerk: Conceived and designed the experiments; Analyzed and interpreted the data; Wrote the paper.

## Funding statement

This work was supported by 10.13039/501100002383King Saud University (6153030450).

## Data availability statement

The data that has been used is confidential.

## Declaration of interest's statement

The authors declare that they have no known competing financial interests or personal relationships that could have appeared to influence the work reported in this paper.

## Appendix A Supplementary material

Multim–a component 1 Multimedia component 1
