# Supplementary material for: Safety and authenticity risks in heritage food preparation at different types of food service establishments: A case study of Saudi Arabia
Source: Heliyon. 2023 Jan 21;9(2):e13042. doi: 10.1016/j.heliyon.2023.e13042 (PMC9922917; doi:10.1016/j.heliyon.2023.e13042)
Supplement: Multim–a component 1 [file mmc1.pdf]

WAGENINGEN UR
For quality of life

The authenticity and safety of heritage food in the food service establishment

Introduction

Dear participant,

Welcome and thank you for agreeing to participate in this survey regarding perception of authenticity and safety risk factors for heritage food.

Heritage foods are increasingly prepared in the food service establishment at a larger scale; which requires more attention to ensure the safety and authenticity of such foods. This survey aims to assess the perception of chefs towards authenticity and safety risk factors of heritage food production in different food service establishments in Saudi Arabia.

The survey consists of five parts:

A. General questions
B. Characteristics of your food service establishment
C. Risk factors of authenticity of heritage food preparation
D. Risk factors of safety in heritage food preparation
E. Closure questions

It will take 10-15 minutes to fill out the whole survey

We want to assure you that your responses are confidential and are completely anonymous. The responses cannot be traced back to the respondent in any way. Besides, your responses will be combined with those of many others and summarised in a report to further protect your anonymity.

Thank you.

Part A. General questions:

1. In which city your restaurant is located?

2. From which region have you cooked heritage food dishes before? (you can tick more than one)

   a. Heritage food dishes from the middle of Saudi Arabia
   b. Heritage food dishes from the south of Saudi Arabia
   c. Heritage food dishes from the north of Saudi Arabia
   d. Heritage food dishes from the west of Saudi Arabia
   e. Heritage food dishes from the east of Saudi Arabia

   Could you give examples of HF dishes below (part of the question)

3. How did you learn to cook the heritage food dishes? (you can tick more than one)

   a. From parents and/or grandparents
   b. Relatives
   c. From social media
   d. From books
   e. At work
   f. Others:

4. For the following statements, please indicate to what extent you agree:

Heritage food is

1. Related to legacy (Food that was cooked in the past and is transferred to the present).
   a. Strongly agree - agree - neutral - disagree - strongly disagree
2. Related to specific people (Native people who cook heritage food dishes).
   a. Strongly agree - agree - neutral - disagree - strongly disagree
3. Related to a specific place (Belong to the geographical area that the heritage dish originates from).
   a. Strongly agree - agree - neutral - disagree - strongly disagree
4. Others explain

Part B. Characteristics of your food service establishment

1. In which food service establishment do you currently work?
    a. Hotels
    b. Heritage restaurants
    c. Catering businesses
    d. Ordinary restaurants (serve heritage food dishes in their menu)

2. How would you characterize your food service establishment?

    a. Private owner
    b. Part of chain
    c. Family owner
    d. Others:

3. Could you indicate the number of native Saudi Arabian workers working in your food service establishment?

    a. None
    b. A few
    c. Some
    d. Most
    e. All are Saudi workers

4. What are your target clients in your food service establishment? (you can tick more than one)

    a. Regional residents
    b. International residents
    c. Local visitors
    d. International visitors

5. Please indicate which regulations/guidelines/standards you use to set up your safety management system? (You can tick more than one)

   a. GHP (Good Hygiene Practices)
   b. HACCP
   c. ISO22000
   d. ISO9001
   e. Others: Explain

6. Which certificate do you have for your food safety management system? (You can tick more than one)

   a. ISO22000
   b. ISO9001
   c. Others:
   d. Not applicable

7. How is the compliance to food safety regulation inspected? (you can tick more than one)

   a. Annual inspection by public authorities
   b. Unannounced inspection by public authorities
   c. Inspection in case of problems by public authorities
   d. Announced third audits to check compliance to the standards
   e. Unannounced third audits to check compliance to the standards
   f. Internal audit
   g. Others: Explain

8. Which measures would be taken in case of non-compliance to the food safety regulations? (you can tick more than one)

   a. Warning
   b. Follow up inspection
   c. Fine
   d. Suspension
   e. End of licenses
   f. Closure of establishment
   g. Others

9. Which measures are taken in case of non-compliance to the private standards (ISO22000, ISO9001, others)? (you can tick more than one)

a. Warning
b. Following-up audits
c. Suspension
d. Certificate not extended
e. Others
f. Not applicable.

Part C. Questions related to authenticity in heritage food preparation

Please indicate to what extent you experienced the following situations when preparing heritage food dishes:

1. Replacing the authentic ingredients because of limited availability.
   always, often, sometimes, rarely, never
2. Replacing the authentic ingredients because of the price.
   always, often, sometimes, rarely, never
3. Adapting the original recipe because of customers' preferences.
   always, often, sometimes, rarely, never
4. Adapting the original recipe because of food safety regulations.
   always, often, sometimes, rarely, never
5. Not following the original cooking procedure step by step because of time constraints.
   always, often, sometimes, rarely, never
6. Not following the original cooking procedure step by step because of limited knowledge and skills.
   always, often, sometimes, rarely, never
7. Using a modernized cooking method and/or equipment instead of traditional ones because of the speed.
   always, often, sometimes, rarely, never
8. Using a modernized cooking method and/or equipment instead of traditional ones because of the food service establishment requirements.
   always, often, sometimes, rarely, never
9. Not authentically presenting the heritage food dishes because of unfamiliarity.
   always, often, sometimes, rarely, never
10. Not following traditional table manners because of unfamiliarity.
    always, often, sometimes, rarely, never
11. Cooking heritage food dishes by non-Saudi chefs due to the lack of experienced local chefs.
    always, often, sometimes, rarely, never

Part D. Questions related to safety in heritage food preparation

Please indicate to what extent you experienced the following situations when preparing heritage food dishes:

1. Purchasing raw ingredients from non-licensed suppliers.
   always, often, sometimes, rarely, never
2. No inspection (visual, smell, dirtiness...etc) of the raw ingredients when receiving them.
   always, often, sometimes, rarely, never
3. Raw ingredients are not immediately stored after receiving.
   always, often, sometimes, rarely, never
4. Raw and processed food are not clearly separated in the fridge/freezers.
   always, often, sometimes, rarely, never
5. Incoming raw ingredients are not systematically labelled.
   always, often, sometimes, rarely, never
6. The storage temperature (refrigerator/freezer) reading exceeds the acceptable range.
   always, often, sometimes, rarely, never
7. Cooking time and temperature is not the same as in the recipe.
   always, often, sometimes, rarely, never
8. Inadequate cleaning of the surfaces for food preparation before re-using.
   always, often, sometimes, rarely, never
9. No cleaning of used equipment for food preparation before re-using.
   always, often, sometimes, rarely, never
10. No separate utensils and cutting boards for preparing food.
    always, often, sometimes, rarely, never
11. No Handwashing before preparing food.
    always, often, sometimes, rarely, never
12. No Handwashing after preparing food.
    always, often, sometimes, rarely, never
13. No washing of hands between tasks (handling raw ingredients, garbage disposal etc).
    always, often, sometimes, rarely, never
14. No wearing gloves during preparing and cooking the food.
    always, often, sometimes, rarely, never
15. Not wearing a hairnet or a cap while preparing and cooking.
    always, often, sometimes, rarely, never
16. Not wearing personal items (e.g. rings, necklaces, watch) while preparing and cooking.
    always, often, sometimes, rarely, never
17. Food handlers touch hair, mouth, and face during food preparation.

always, often, sometimes, rarely, never

18. Employees work when they have illnesses.
    always, often, sometimes, rarely, never

Part E: Closure questions:

1. What is your gender?
   a. Male
   b. Female
2. What is your age?

3. What is your education level?
   a. Below secondary school
   b. Secondary school
   c. Diploma
   d. Bachelor
   e. Postgraduate degree or above

4- How many years of experience do you have working in the food service establishment?

5- How many years of experience do you have in cooking heritage dishes?

6- What type of food safety training did you follow? (You can tick more than one)

a. I had no a particular food safety training
b. I had basic food safety training in the restaurant
c. I had official food safety training (HACCP, ISO22000, others).
d. I had food safety courses in education (university).
e. Others:

- If yes, please provide your email and telephone number below.
  Email:
  Mobile number:

The authenticity and safety of heritage food in food service establishments

This is the end of the survey

Thank you for participating in this survey!

If you have any questions, please contact me!

Mohammad Almansouri | PhD Candidate

E-mail: mohammad.almansouri@wur.nl

Wageningen University & Research
AFSG – Food Quality and Design
P.O. Box 17, 6700 AA Wageningen
Bornse Weilanden 9, 6708 WG Wageningen
Wageningen Campus l Building 118 (Axis)

www.wur.nl
